# The effects of vitamin D_2_ or D_3_ supplementation on glycaemic control and related metabolic parameters in people at risk of type 2 diabetes: protocol of a randomised double-blind placebo-controlled trial

**DOI:** 10.1186/1471-2458-13-999

**Published:** 2013-10-23

**Authors:** Ravi K Menon, Anna P Rickard, Nasima Mannan, Peter M Timms, Stephen J Sharp, Adrian Martineau, Barbara J Boucher, Tahseen A Chowdhury, Christopher J Griffiths, Simon J Griffin, Graham A Hitman, Nita G Forouhi

**Affiliations:** 1 Blizard Institute, Queen Mary University of London, London, UK; 2 Medical Research Council Epidemiology Unit, University of Cambridge, Cambridge, UK; 3 Homerton University Hospital NHS Foundation Trust, London, UK; 4 Barts Healthcare NHS Trust, London, UK; 5 Centre for Health Sciences, Queen Mary University of London, London, UK; 6 Blizard Institute, Barts and The London School of Medicine and Dentistry, 4 Newark Street, London E1 2AT, UK

**Keywords:** Vitamin D_2_, Vitamin D_3_, Placebo, Type 2 diabetes, Randomised, Trial, Intervention

## Abstract

**Background:**

The global prevalence of type 2 diabetes is increasing. Effective strategies to address this public health challenge are currently lacking. A number of epidemiological studies have reported associations between low concentrations of 25-hydroxy vitamin D and the incidence of diabetes, but a causal link has not been established. We investigate the effect of vitamin D supplementation on the metabolic status of individuals at increased risk of developing type 2 diabetes.

**Methods/design:**

In a randomised double-blind placebo-controlled trial individuals identified as having a high risk of type 2 diabetes (non-diabetic hyperglycaemia or positive diabetes risk score) are randomised into one of three groups and given 4 doses of either placebo, or 100,000 IU Vitamin D_2_ (ergocalciferol) or 100,000 IU Vitamin D_3_ (cholecalciferol) at monthly intervals. The primary outcome measure is the change in glycated haemoglobin level between baseline and 4 months. Secondary outcome measures include blood pressure, lipid levels, apolipoproteins, highly sensitive C-reactive protein, parathyroid hormone (PTH) and safety of supplementation. and C-reactive protein. The trial is being conducted at two sites (London and Cambridge, U.K.) and a total of 342 participants are being recruited.

**Discussion:**

Trial data examining whether supplementation of vitamin D improves glycaemic status and other metabolic parameters in people at risk of developing type 2 diabetes are sparse. This trial will evaluate the causal role of vitamin D in hyperglycaemia and risk of type 2 diabetes. Specific features of this trial include recruitment of participants from different ethnic groups, investigation of the relative effectiveness and safety of vitamin D_2_ and D_3_ and an evidence based approach to determination of the dose of supplementation.

**Trial registration:**

EudraCT2009-011264-11; ISRCTN86515510

## Background

The number of people with diabetes is increasing across the world, with the latest estimates from the International Diabetes Federation predicting a rise from 366 million in 2011 to 552 million in 2030 [1]. The clinical consequences of type 2 diabetes (T2D) including macrovascular and microvascular disease, and related premature mortality [2] make it a condition of major public health importance. As such, the identification of risk factors for T2D, particularly those that might be potentially modifiable, is of great interest.

There is convincing evidence that changes to diet and physical activity can help to prevent or delay the onset of T2D among those at high risk [3-6]. However, intensive lifestyle modification interventions are difficult to sustain and costly to implement. Thus, there is interest in identifying complementary strategies that might help in the prevention of T2D. There is accumulating evidence that vitamin D insufficiency is associated with the risk of developing T2D [7-9]. Whether supplementation of vitamin D can delay or prevent T2D is unknown.

Epidemiological studies have examined the association between dietary vitamin D intake and the risk of diabetes [10,11], but these omitted information on the major non-dietary component of vitamin D from endogenous synthesis through sun exposure. Circulating 25-hydroxyvitamin D (25[OH]D) concentration is the best indicator of vitamin D status as it accounts for both vitamin D intake and endogenous synthesis [12]. More recent epidemiological studies have examined the association between circulating 25(OH)D concentrations and incident diabetes, and the findings have been largely consistent, demonstrating an inverse association. Such findings have been summarised in systematic reviews and meta-analyses [7-9], with the latest one from our group showing a 41% (95% confidence interval 33% to 48%) lower risk for those in the highest versus the lowest quartile of 25(OH)D [7]. Whilst meta-analysis deals with issues of consistency and precision, it does not resolve the problems of confounding and selection bias in observational studies. To address the question of whether observed associations between vitamin D and risk of T2D are causal in nature, there is a need to conduct randomised controlled trials of supplementation.

Two clinical trials that examined incident diabetes as an outcome found no effect of supplementation with 400 IU/day and 800 IU/day of vitamin D respectively [13,14]. In the larger of these trials (the Women’s Health Initiative randomised controlled trial), there was no association between vitamin D supplementation (in combination with calcium supplementation) and T2D incidence (hazard ratio 1.01, 95% CI: 0.94; 1.10) [13]. A causal link could not be ruled out by this trial, however, as it tested a low dose of 400 IU/day of vitamin D_3_[15]. In two separate systematic reviews, Mitri and Pittas et al. synthesised the available evidence on clinical trials of the effect of vitamin D supplementation on diabetes incidence (8 trials) or intermediate outcomes such as levels of glycaemia (11 trials) [8,9]. Overall, few were considered of good quality, but there was no overall benefit of supplementation with vitamin D. Of five trials measuring insulin resistance as an outcome [8], only one in New Zealand among vitamin D deficient (25(OH)D< 50 nmol/l) South Asian women reported significant improvement assessed by homeostasis model assessment [16]. More recently, Pittas’ group investigated whether vitamin D supplementation, with or without calcium, improved glucose homeostasis in adults at high risk of diabetes [17]. They included 92 adults (mean BMI 32 kg/m^2^, mean glycated haemoglobin of 5.9% (41 mmol/mol)) who were randomly assigned in a 2-by-2 factorial-design trial, to receive either cholecalciferol (2000 IU once daily) or calcium carbonate (400 mg twice daily) for 16 weeks. They reported improved beta cell function in the vitamin D supplemented group with increased glucose disposition index and improved insulin secretion. There was no significant effect on glycated haemoglobin (HbA_1c_) level, although there was a tendency toward greater attenuation in the rise in HbA_1c_ over follow up. There was also no significant effect of calcium supplementation. Two further trials have since been published showing no associations between supplementation with relatively high dose vitamin D and markers of insulin sensitivity [18,19]. These trials were restricted to minority groups (Latin Americans or African Americans) or women, and a common feature was the successful increase in 25(OH)D level following D_3_ supplementation. In the Davidson et al. study [18], there was also no effect on diabetes incidence, but follow up was short at 12 months. However, at 12 months, HbA_1c_ levels were significantly lower in the vitamin D group compared with the placebo group. Taken together, the evidence to date from trials is suggestive but not conclusive, and there is further need for well-designed trials, with adequate sample size, adequate dose of supplementation and good adherence, to advance this field of enquiry. Three particular issues are of further interest. The first relates to the dose of supplementation. Recent Institute of Medicine (IOM) guidelines suggested that the vitamin D needs of at least 97.5% of the population would be met by a recommended dietary allowance (RDA) of 600 IU/day for adults aged up to 70, and 800 IU/day after the age of 71 years [20]. IOM also raised the safe limit of vitamin D intake to 4000 IU/day and suggested that a serum concentration of 50 nmol/l is sufficient for most people [21]. However, several authors have suggested that IOM recommendations are focussed on bone health alone, do not account for potential extra-skeletal effects and are therefore too conservative [22]. A growing body of evidence suggests that larger doses than those previously investigated (equivalent to 2,000 IU – 10,000 IU daily) are required to optimise vitamin D status [15].

A second issue relates to whether vitamin D_2_ (ergocalciferol) or vitamin D_3_ (cholecalciferol) is likely to be more effective, or whether they would have equivalent effects on glycaemia. Previously thought to be equipotent in elevating serum 25(OH)D, several studies now suggest that vitamin D_2_ is less effective than vitamin D_3_[23-27]. In contrast, a trial by Hollis et al. reported that vitamin D_2_ and D_3_ were equivalent in their ability to maintain circulating levels of 25(OH)D over 11 weeks of supplementation [28]. Most recently, based on a meta-analysis of clinical trials that directly compared the 25(OH)D raising effects of supplementation with D_2_ or D_3_, Tripkovic et al. concluded that D_3_ supplementation is more efficacious [29]. However, according to the free hormone hypothesis, despite being less efficacious in elevating total 25(OH)D levels, 25(OH)D_2_ may be more available to exert biological activity being less tightly protein bound than 25(OH)D_3_. However, the effects of factors such as hydroxylation rates and deactivation of D_2_ and D_3_ on biological activity are unclear, and no trial has yet reported on the comparative effects of D_2_ and D_3_ on glycaemia.

A further issue relates to the effectiveness of vitamin D supplementation in people from different ethnic groups. Melanin in the skin reduces vitamin D production and hence the darker the skin pigmentation, the lower the synthesis of vitamin D for equivalent sun exposure [30]. In a study in the United States, researchers exposed healthy young individuals from different races to similar doses of UVB rays and measured the 25(OH)D concentrations before and after exposure. Baseline concentrations were similar, but Caucasians had highest concentrations of 25(OH)D following UVB exposure, followed by East Asians, South Asians and Blacks [31]. Concentrations of 25(OH)D in Hispanics and African-Americans are lower than those among the Caucasians [32]. Ethnic groups in the UK such as the South Asian population, have especially high rates of hypovitaminosis D [33-35]. It is therefore important to examine the effectiveness of vitamin D supplementation on levels of glycaemia among people of different ethnic groups in the same study, to add to the literature on this topic [16,18,19,36].

We aim to address these unresolved issues, especially concerning the nature of the association between vitamin D and metabolic parameters among people at risk of diabetes. We particularly wanted to include men and women of different ethnic groups, evaluate effects of an adequate dose of supplementation, and test the relative efficacy of vitamin D_2_ and D_3_ versus placebo among those at elevated risk of diabetes. The results obtained should provide high quality scientific evidence to improve understanding, and will also help to inform the conduct of larger trials with disease end points such as diabetes and cardiovascular disease.

### Objectives

The primary objective of this trial is to determine whether oral supplementation with vitamin D_2_ or vitamin D_3_ can lead to reduction in glycaemia and an improvement in related metabolic abnormalities in people at risk of developing diabetes.

The secondary objectives of this trial are (i) to examine the feasibility and acceptability of vitamin D supplementation to inform the design of a future RCT with diabetes and/or cardiovascular endpoints, and (ii) to compare the efficacy of vitamin D_2_ and vitamin D_3_.

### Sponsors

The trial is jointly sponsored by Queen Mary University of London and the Medical Research Council Epidemiology Unit at Cambridge. Trial funding is from a block grant from the NHS Tower Hamlets and from MRC Epidemiology Unit core funding (MC_UP_A100_1003).

## Methods/design

We designed a double-blind, placebo-controlled randomised clinical trial amongst people at risk of developing T2D, across two sites - East London and Cambridge, UK. We are randomly allocating 342 participants to one of three groups, to receive a monthly dose for four months, of 100,000 IU (equivalent to 2.5 mg) of either cholecalciferol (vitamin D_3_) or ergocalciferol (vitamin D_2_) or a monthly dose of placebo (miglyol oil). Each participant is followed-up for a total of 4 months from their first visit. The inclusion and exclusion criteria of the trial are as follows.

### Inclusion criteria

Men and women aged 30–75 years, from any ethnic group, who can provide informed consent for participation in the trial and are at a high risk of developing type 2 diabetes are included.

Risk of developing diabetes is defined by the following criteria:

A. Non-diabetic hyperglycaemia; either impaired glucose tolerance (IGT) or impaired fasting glucose (IFG) as defined by current World Health Organisation (WHO) criteria [37], or HbA_1c_ between 5.5% and 6.49% (equivalent to 37 to 47 mmol/mol), where this information is available in medical records (London), or in the records of studies in which the participants have consented to being re-approached to consider participating in future studies (Cambridge), or,

B. Cambridge Risk Score (CRS) [38-41] cut offs that indicate increased risk for diabetes. The CRS cut-offs are 0.236 for the Black/Caribbean population, 0.127 for South Asians and 0.199 for Caucasians [39]. For other groups the cut-off for Caucasians is used. This approach is used at the London site.

### Exclusion criteria

Participants are excluded if they have a known history of diabetes or have used oral hypoglycaemic agents, have random blood glucose during initial screening >11 mmol/l, have known intolerance to vitamin D_2_ or D_3_ within the previous two months or are currently using vitamin D supplements, have a known history of hypercalcaemia (serum calcium >2.65 mmol/l) or point of care ionised calcium >1.3 mmol/l. They are also excluded if they have known stage 4 or worse chronic kidney disease (eGFR (estimated glomerular filtration rate) < 30 ml/min), a history of significant liver disease (AST (aspartate aminotransferase) >3 x upper limit of normal (ULN) or ALT (Alanine aminotransferase) >3 x upper limit of normal (ULN) or serum bilirubin > 2.5 x ULN), a known history of renal stones, hyperparathyroidism, active sarcoidosis, tuberculosis or malignancy (active defined as currently on treatment and/or medication for the above conditions), have taken cardiac glycosides or oral/ intramuscular/intravenous corticosteroids (excluding inhaled and topical corticosteroids) in the past one month. They are excluded if they have known current anaemia (haemoglobin <11 g/dL) or haemoglobinopathy such as sickle cell anaemia and beta or alpha thalassemia. Additionally, if they plan to travel out of the London or Cambridge area (depending on site of recruitment) within 8 weeks of enrolment such that it would disrupt monitoring of the participant, they are excluded. Among women, current breast feeding, pregnancy or planning a pregnancy are also considered exclusion criteria.

### Outcomes

The primary endpoint of the trial is HbA_1c_. There are multiple secondary endpoints including systolic and diastolic blood pressure, random cholesterol, HDL cholesterol, ApoA1 and ApoB apolipoproteins, CVD risk score as assessed by the UKPDS risk engine (version 2) [42] and additionally in London only, a measurement of arterial stiffness assessed by pulse wave velocity. Further secondary endpoints include anthropometry (waist circumference and body mass index) and serum concentrations of C-reactive protein (measured using a high sensitivity assay), fructosamine, and parathyroid hormone. Other endpoints include the safety of oral vitamin D without a pre-assessment of vitamin D status, the proportion of participants with a serum 25(OH)D in categories of <25, 25 to <50, 50 to <75, 75 to <150 and greater than or equal to 150 nmol/L measured by an LC-MS/MS (liquid chromatography–tandem mass spectrometry) assay, and the feasibility and acceptability of the intervention. The trial also assesses functional status (SF-8), health utility (EQ-5D) and total body pain (BPI).

### Intervention

In this trial the dose of vitamin D being used is 100,000 IU (equivalent to 2.5 mg) per month administered as four oral doses over consecutive months. The two investigational medicinal products (IMP) include cholecalciferol (prepared as Vigantol oil containing 20,000 IU vitamin D_3_ per ml in Miglyol vehicle oil), or ergocalciferol (prepared as Sterogyl containing 20,000 IU vitamin D_2_ per ml in ethanol). In the active intervention groups this represents a daily dose equivalent of ~3,300 IU. The placebo is Miglyol 812 oil with esters of coconut and palm-derived oils.

We chose the oral route of administration as this is the most commonly used in clinical practice, and it results in less inter-individual variability in achieved serum 25(OH)D concentrations compared to the intramuscular route [43]. It is also the route used by other investigators in studies which show safety of the dosage regimen used in this trial [44,45].

#### Investigational medicinal products

Vigantol oil (cholecalciferol) is manufactured by Merck Serono GmbH in the Federal Republic of Germany at a licensed site under GMP (Good Manufacturing Practices, WHO) conditions. Sterogyl (ergocalciferol) is manufactured by DB Pharma in France at a licensed site, also under GMP conditions. The placebo (‘Miglyol oil’), which is also the vehicle for vitamin D_3_ in Vigantol oil does not contain any pharmacologically active ingredient. The investigational agents are repackaged into randomisation packs by a commercial laboratory (Nova Laboratories).

### Safety

The vitamin D investigational medicinal products used in this trial have undergone safety and quality checks, and are prescribed by a registered medical practitioner in both study centres. We wanted to use an adequate dose of vitamin D and after careful consideration opted for a dose of 100,000 IU per month, in order to increase the likelihood of generating a significant difference in vitamin D status between the treatment and placebo groups. There have been several trials in which high doses of vitamin D were administered without any adverse events. In an Indian randomised controlled trial, supplementation in men with three doses of 120,000 IU of vitamin D at fortnightly intervals was not associated with any adverse events in general or specifically relating to hypercalcaemia [36]. In a Canadian trial, 4000 IU of vitamin D_3_ daily for 2–5 months in healthy adults did not result in any adverse effects [46]. Two clinical studies of 10,000 IU/day have demonstrated an elevation of serum 25(OH)D concentrations to the high end of normal concentrations without inducing toxicity in healthy volunteers [44,45]. The recent IOM (Institute of Medicine) aforementioned review revised the safe dose of vitamin D upwards from 2000 IU/day to 4000 IU/d, which the dose we are supplementing falls well within [21]. Side effects of vitamin D supplementation are rare and include gastrointestinal symptoms (such as nausea, constipation, or diarrhoea), hypercalcaemia, hypercalcuria and associated kidney stones. To minimise risk of hypercalcaemia and hypercalcuria amongst predisposed individuals, we exclude individuals with known sarcoidosis, malignancies and tuberculosis. As a safety precaution, three measures are recorded for all recruited participants at all trial visits: a point of care ionised calcium test, a laboratory serum corrected calcium test and a laboratory urinary calcium to creatinine ratio test. Trial participants who have high point of care ionised calcium (>1.3 mmol/L) or urine calcium:creatinine ratio (molar ratio >1) or serum corrected calcium > 2.65) are excluded from further doses of the IMP, but continue to be followed up. Additionally, provision has been made for the recording of adverse events or reactions.

### Recruitment

#### London site

Electronic searches of lists of patients in GP practices in East London are done by the practice staff to identify suitable participants. The Cambridge Risk Score (CRS) [38-41] is used to identify participants who are at high risk for T2D. In addition, a previous record of the presence of impaired glucose tolerance, impaired fasting glucose or non-diabetic hyperglycaemia assessed by HbA_1c_ is used to identify potential eligible participants. Practice staff extract information from the medical records on the following variables which comprise the Cambridge Risk Score: age, sex, smoking status, family history of diabetes, body mass index and whether the patient is prescribed anti-hypertensive or steroid medication. In addition, they extract information on ethnicity. Once identified, and after applying exclusion criteria that can be accessed from medical records, potential participants receive a letter inviting them to participate in the trial, a reply slip, a patient information sheet and a flyer with contact information. Potential participants who reply expressing an interest are telephoned by a member of the study team to provide further information as required and to arrange an appointment to attend a research centre.

### Cambridge site

In Cambridge, participants are recruited from the Fenland study, an on-going population-based observational study investigating the influence of lifestyle and genetic factors on the development of diabetes, obesity, and other metabolic disorders [47]. Residents of Cambridgeshire, in the east of England, born between 1950 and 1975 are potentially eligible to participate in the Fenland Study and are excluded by their general practitioner if they have been diagnosed with diabetes or a terminal illness with a prognosis of less than one year, have a psychotic illness, are pregnant or lactating, or if they are unable to walk unaided. Approximately 28% of those registered with participating general practices in the Cambridgeshire Primary Care Trust have enrolled in the Fenland Study (more than 8,000 participants). Fenland study participants are eligible for this trial if they had agreed to be contacted again to gauge interest in involvement in future studies and do not meet the trial exclusion criteria. Potential participants are mailed an invitation letter for the trial, an information sheet and a flyer with contact information. Those interested in taking part are asked to complete the response form and to return it in an enclosed freepost reply envelope. In the event of non-response, a second mailing is sent approximately four weeks after the first. Responders are excluded from the trial if they have been diagnosed with diabetes or are actively participating in another trial, or if any of the trial exclusion criteria apply. For eligible participants, telephone contact is made and any queries are addressed before setting an invitation date for the baseline visit. In Cambridge, HbA_1c_ is primarily used as the eligibility criterion for identifying increased risk of diabetes.

### Randomisation and blinding

Randomisation into three groups (cholecalciferol (D_3_), ergocalciferol (D_2_), or placebo) follows stratification of participants on the basis of age (two groups 30–50 and 51–75 years) and sex (male and female) into four groups, with a block size of 6 within each group.

In this double-blind trial, neither the participant, the investigators, nor the laboratory staff know whether participants have been allocated to vitamin D_2_, D_3_ or placebo during the course of the study. Since the IMPs are oil based or ethanol based products, in order not to compromise allocation concealment, the trial drug is administered by a person other than the investigator. The participants are also asked not to let the investigator know about any physical characteristics (including taste and feel) of the product that they have consumed.

### Trial procedures and follow-up

**Figure 1:**
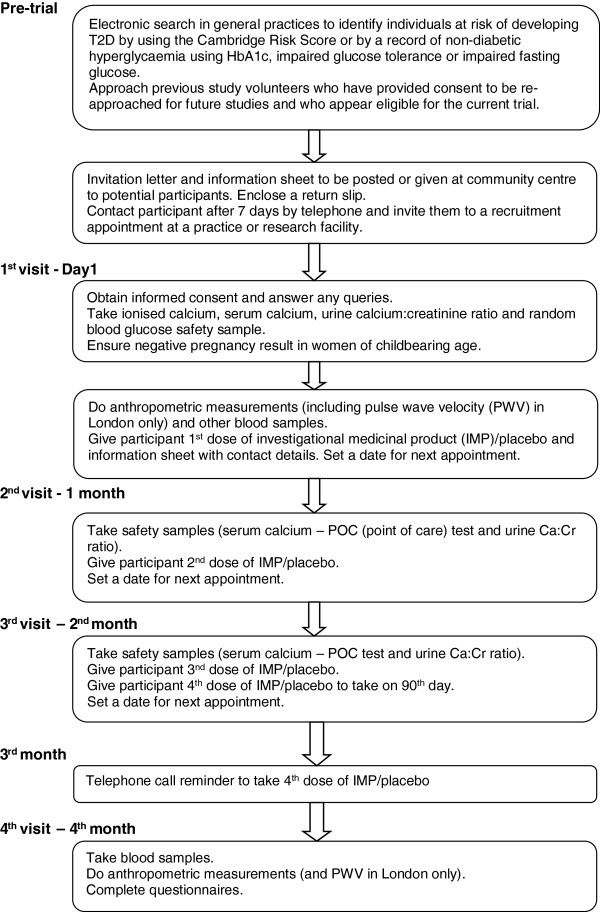
Trial visits and procedures for participants.

As shown in the Figure 1, each participant has a total of 4 visits to the trial centre and one telephone call at 3 months to discuss the appropriateness of taking the final of the four doses of IMP. The initial visit includes informed consent, assessment of eligibility, administration of questionnaires regarding physical activity, diet, sun exposure, functional status, health utility, and a brief pain inventory, randomisation and administration of the first dose of study IMP. Subsequent visits (and the telephone call) are at monthly intervals. Second and third visits are for safety assessment and observation of consumption of the next two doses of the study IMP, while the 4^th^ (last) dose of the IMP is taken at home prompted by a phone call from the trial staff, if prior safety checks are within normal limits. The final follow-up visit is at the end of 4 months, when questionnaires are repeated and a final set of blood samples are collected.

### Blood collection and assays

The trial team collects baseline blood samples from all participants during the first visit, after informed consent, to assess levels of serum ionised calcium as well as serum 25(OH)D assay, HbA_1c_ and the other secondary endpoints mentioned earlier. During the second and third visits blood samples are collected only for safety analysis (as described under the section on safety) to make a judgement about continuation of the IMP. During the final (fourth) visit all blood samples are repeated, as during the first visit. Assays for safety monitoring (serum ionised calcium) at all visits are performed contemporaneously. For other trial endpoint assays, the HbA_1c_ samples from the first and fourth visit are analysed immediately on fresh samples, while aliquots for all other assays (including 25(OH)D) are stored frozen at -70°C to be measured at the end of the trial. Thus in summary, blood samples for safety are collected at all four visits, while for other endpoints bloods are collected only at the first (baseline) and fourth (final) visit.

HbA_1c_, the trial primary end-point is measured according to IFCC (International Federation of Clinical Chemistry and Laboratory Medicine) standards in both trial centres (Royal London Hospital Biochemistry Laboratory for East London and Addenbrookes Hospital Biochemistry Laboratory for Cambridge). Results are reported in both IFCC and DCCT units. Serum 25(OH)D measurement is performed at Homerton University Hospital NHS trust laboratory (London). This laboratory participates in the DEQAS quality assurance scheme and measures both 25(OH)D_2_ and D_3_ using the LC/MS/MS (liquid chromatography/ mass spectrometry/mass spectrometry) method which is well validated [48]. The other measurements including parathyroid hormone, fructosamine, hsCRP and lipid assays are also carried out at Homerton University Hospital NHS Trust laboratory.

### Sample size

Sample size calculations showed that 207 participants (69 per randomised group) are required to detect a 0.2% difference in mean HbA_1c_ between the placebo and either vitamin D_2_ or vitamin D_3_ groups (power calculation was based on DCCT units) with 90% power and a 5% significance level, assuming a standard deviation of HbA_1c_ of 0.5%. However, given the importance of the secondary endpoint systolic blood pressure (SBP), a further calculation was performed to estimate the sample size that would be needed to detect a 5 mmHg difference in mean SBP between randomised groups, assuming a standard deviation of 16 mmHg. The assumptions for the sample size calculations were based on data from the ProActive trial [49] and the BanglaDip Study (personal communication). This yielded a requirement of 339 participants (113 in each group). Pragmatically, recruiting this larger number of participants will also mean that the study will still be powered for the primary endpoint (HbA_1c_) even if there are losses to follow up. Because 339 is not divisible by 6 (the block size for randomisation), the target number is 342 across the two test sites.

### Statistical analysis plan

The analysis plan is available at http://www.mrc-epid.cam.ac.uk/files/2013/04/VitDtrial_AnalysisPlan_Sep2012.pdf. In brief, the primary analysis of efficacy endpoints uses an Intention-To-Treat (ITT) population, which includes all participants for whom outcome data are available, in the group to which they were randomised, regardless of the treatment actually received. A secondary analysis of efficacy endpoints uses a Per-Protocol (PP) population. This population excludes individuals who did not comply with the protocol (e.g. individuals who did not take all doses of the IMP). The analysis of safety endpoints uses a safety population, which includes all participants in the group based on treatment actually received. Any individual who received at least one dose of either vitamin D_2_ or vitamin D_3_ will be included in the vitamin D_2_ or vitamin D_3_ group.

Baseline characteristics of the study population will be summarised separately within each randomised group. For continuous variables, means and standard deviations will be presented, unless the variable has a highly skewed distribution, in which case, medians, 25^th^ and 75^th^ percentiles will be presented. For categorical variables, the number and percentage of participants within each category will be presented. For each variable (continuous or categorical), the percentage of missing values will be reported. No p-values will be calculated for these tables.

The primary efficacy endpoint, HbA_1c_, will be compared separately between each treatment group and placebo, using analysis of covariance with adjustment for baseline and centre. Where baseline values are missing, the missing indicator method [50] will be used to enable these participants to be included in the analysis. For both vitamin D_2_ vs. placebo and vitamin D_3_ vs. placebo, the difference in mean HbA_1c_, 95% confidence interval and p-value will be reported. An analysis will be performed to check whether adjusting for age and sex (the stratifiers) in the analysis of covariance model has any impact on the estimated treatment effects; if it has no impact, then they will not be included in the model. HbA_1c_ will be analysed and results will be reported in IFCC units (mmol/mol), and to enable those readers who are still using the DCCT% units, we will also provide equivalent results using the DCCT% units, derived using a previously described conversion formula [51].

For each of the secondary efficacy endpoints, differences in means (for continuous endpoints) or proportions (for binary outcomes) between each treatment group and placebo, together with 95% confidence intervals, will be estimated using the same method described for the primary endpoint. Continuous endpoints with skewed distributions will be log transformed prior to analysis, in which case a ratio of geometric means (and confidence intervals) will be reported.

The number and percentage of participants experiencing any of the safety endpoints will be reported separately within each randomised group, without p values. For the primary endpoint, interactions between treatment group and baseline HbA_1c_, and treatment group and baseline vitamin D will be tested by including the appropriate interaction term in the analysis of covariance model. If the p-value for the interaction test is <0.05, the treatment effects and 95% confidence intervals will be estimated within subgroups defined by levels above and below the median value of either HbA_1c_ or vitamin D.

For the primary efficacy endpoint, two p-values will be calculated, one for the comparison of vitamin D_2_ vs placebo and one for the comparison of vitamin D_3_ vs placebo. No adjustment for multiple testing will be performed. An exploratory analysis will be performed in which mean HbA_1c_ will be compared between the vitamin D_2_ group and the vitamin D_3_ group, using the same method described above. An estimate of the difference in mean HbA_1c_ (adjusted for baseline), together with a 95% confidence interval, will be reported. For all other efficacy endpoints, the treatment effects (vitamin D_2_ vs placebo and vitamin D_3_ vs placebo) will be reported together with a 95% confidence interval. No p-values will be calculated. Interpretation of results for secondary endpoints will be cautious and results that are statistically significant in isolation will be interpreted less strongly than sets of results that are mutually supportive, or which are supported by previous research findings.

### Trial monitoring

An independent data monitoring committee (DMC) and trial steering committee (TSC) has been set up to monitor the trial. This consists of experts in biostatistics, diabetes, clinical trials and biochemistry. The TSC includes a lay member to ensure that the views of potential participants concerning the trial design and conduct are represented. The DMC and TSC will monitor progress of the trial, adverse event data and will recommend continuation, modification or early termination of the trial. Decisions will also be influenced by any other relevant trial data available during the trial. No interim analysis is being planned.

### Data management and quality assurance

For the Cambridge site, each participant is assigned a unique numeric identifier at the beginning of the Fenland Study, and a new identifier is assigned to participants enrolled in the vitamin D supplementation trial. For the London site, a unique numeric identifier is assigned to each trial participant. All personal data are stored on an encrypted drive, and links to personal information are only available to the study coordination team. Consent forms and questionnaire data are stored in locked filing cabinets in secure protected sites. Questionnaire data are double entered by an independent, quality assured data-entry company.

### Ethics

Ethical approval for the trial was provided by the Charing Cross Medical Ethics Committee (reference no 09/H0711/85; on 22^nd^ December 2009). For the Cambridge site, full ethical approval for the Fenland Study was obtained from the Cambridge Local Research Ethics Committee on the 11th of May 2004 (reference number 04/Q0108/19). At both sites, written informed consent is obtained from all participants, and each participant’s general practitioner is notified of their enrolment.

## Discussion

There is widespread interest in the potential role of vitamin D in the prevention of diabetes and related complications, but despite an increasing number of publications on this topic, the potential causal association between vitamin D and type 2 diabetes has not been confirmed. It is tantalising to speculate that if there is a causal association the population health impact of vitamin D supplementation could be substantial, as we previously estimated a population attributable fraction of nearly 18% associated with 25(OH)D levels in the insufficiency range (<50 nmol/l) [7]. Achieving and sustaining lifestyle modification for the prevention of diabetes, though efficacious [6] is challenging, and hence the potential role of optimal vitamin D status in reducing diabetes burden is attractive.

This trial has several strengths: the inclusion of adults from different ethnic groups, of varying ages and both sexes, the decision to use a relatively high dose of vitamin D supplementation at the daily equivalent dose of 3,300 IU per day that should be effective in raising 25(OH)D levels, and the comparison of both vitamin D_3_ and vitamin D_2_ against placebo, combined with supervised bolus dosing. Following the principles of Good Clinical Practice (GCP) we have placed participant welfare as a critical consideration and have applied rigorous safety checks and adverse event monitoring. We are using the well validated and quality assured method for measuring 25(OH)D, with tandem mass LC-MS/MS spectrometry. The secondary endpoints will also enable the examination of assessment of modelled cardiovascular risk, and of the feasibility and safety of 25(OH) in relatively high doses. The limitations of the trial merit consideration. The study sample size allows comparison of D_2_ against placebo and D_3_ against placebo, and while we will conduct an exploratory analysis of D_2_ against D_3_, the study is not large enough to address this question. The trial follow-up period is 4 months, and whilst changes in HbA_1c_ take around 6 weeks to happen, it can be argued that the follow-up period is too short. To mitigate this effect we are also measuring fructosamine which is more sensitive to changes in levels of blood glucose over a shorter period. We are not measuring fasting glucose and insulin and will therefore not be able to estimate insulin resistance. This decision was made *a priori*, to enhance participation and is in keeping with the decision around the time of trial inception that HbA_1c_ can be used as a diagnostic criterion for diabetes [52]. Finally, this trial is measuring markers of glycaemia (e.g. HbA1c) and of cardiovascular risk (e.g. pulse wave velocity, modelled CVD risk) without ‘hard’ endpoints of incident events of diabetes or cardiovascular disease. While the current endpoints are important in their own right to understand the effects of vitamin D supplementation on these parameters, and indeed we have included adequate sample size to enable us to investigate this appropriately, it will also be important to assess clinical events in specifically designed future trials.

The results of this trial should contribute robust evidence concerning whether supplementation with vitamin D improves glycaemic markers and related metabolic disturbances.

## Abbreviations

ALT: Alanine Transaminase; AST: Aspartate Transaminase; BMI: Body Mass Index; BPI: Body Pain Index; CRP: C Reactive Protein; CRS: Cambridge Risk Score; CVD: Cardio-Vascular Disease; DCCT: Diabetes Control and Complications Trial; DMC: Data monitoring committee; eGFR: Estimated Glomerular Filtration rate; GCP: Good Clinical Practice; GMP: Good Manufacturing Practices; HbA1c: Glycated Haemoglobin; HDL: High Density Lipoprotein; hsCRP: high-sensitivity C Reactive Protein; IFCC: International Federation of Clinical Chemistry and Laboratory Medicine; IFG: Impaired Fasting Glucose; IGT: Impaired Glucose Tolerance or; IMP: Investigational Medicinal Product; IOM: Institute of Medicine; ITT: Intention-To-Treat; LC-MS/MS: Liquid Chromatography–tandem mass spectrometry; LDL: Low Density Lipoprotein; MRC: Medical Research Council, UK; NHS: National Health Service, UK; PP: Per-Protocol; RCT: Randomised Controlled Trial; T2D: Type 2 Diabetes; TSC: Trial Steering Committee; UKPDS: United Kingdom Prospective Diabetes Study; ULN: Upper limit of normal; WHO: World Health Organisation.

## Competing interests

None of the authors have any competing interests to declare.

## Authors’ contributions

All authors participated in the design of the study and SJS helped with the statistical analysis plan. GAH was chief investigator and NGF the lead investigator, and GAH, SJG, NGF, RM, NM and APR were responsible for the conduct and monitoring of the trial. PMT advised on laboratory measurements, AM, BJB, TAC and CJG advised on trial related issues. RM and NM conducted data collection in London and APR helped with study coordination at Cambridge. RM and NGF drafted the manuscript. All authors gave intellectual input and read and approved the final manuscript.

## Pre-publication history

The pre-publication history for this paper can be accessed here:

http://www.biomedcentral.com/1471-2458/13/999/prepub
